# Native yeast kinetochore structures identify an essential inner kinetochore interaction

**DOI:** 10.64898/2026.01.30.702844

**Published:** 2026-01-31

**Authors:** Mengqiu Jiang, Changkun Hu, Sabrine Hedouin, Angelica Andrade Latino, Yasuhiro Arimura, Andrew B. Stergachis, Sue Biggins

**Affiliations:** 1 Howard Hughes Medical Institute, Division of Basic Sciences, Fred Hutchinson Cancer Center, Seattle, WA, United States of America; 2 Division of Basic Sciences, Fred Hutchinson Cancer Center, Seattle, WA, United States of America; 3 Division of Medical Genetics, Department of Medicine, University of Washington, Seattle, WA, United States of America; 4 Department of Genome Sciences, University of Washington, Seattle, WA, United States of America

**Keywords:** Chromosome segregation, Kinetochore, Centromere, DNA bending, Ndc10

## Abstract

Kinetochores must accurately assemble on centromeres every cell cycle for faithful chromosome segregation. Although a conserved centromeric histone variant is essential for inner kinetochore formation, the budding yeast centromeric DNA is a poor template for nucleosome formation *in vitro*, possibly due to a resistance to bend. To better understand how the yeast inner kinetochore is assembled, we developed a one-step protocol to purify *de novo* assembled native inner kinetochore subcomplexes for structural studies. We performed cryoelectron microscopy on the purified complexes and generated medium to high resolution density maps of four separate inner kinetochore complexes, two of which had not previously been visualized. We detected differences between native and previously reconstituted structures, suggesting that the *de novo* assembly assay generated intermediate assemblage states. A strong extra structural density, which corresponds to an Ndc10 trimerization domain, associated with centromeric DNA and a pair of CBF3 complexes to induce significant centromere bending. Its deposition on the CBF3-CEN complex is essential for kinetochore assembly and chromosome segregation. We propose that Ndc10 trimerization facilitates bending of the centromeric DNA, leading to assembly and stabilization of the centromeric nucleosome and inner kinetochore.

## Introduction

Faithful chromosome segregation ensures that replicated chromosomes are evenly distributed to daughter cells. Chromosome segregation is mediated by the kinetochore, a conserved protein machine that interacts with microtubules to separate chromosomes during cell division^1,2^. The kinetochore assembles on centromeres, chromosomal loci that are usually composed of thousands to millions of repetitive DNA base pairs that are not sequence-defined^3^. Centromeres are therefore epigenetically specified by specialized centromeric nucleosomes containing a histone H3 variant called CENP-A^4,5^.

**Figure 1.**
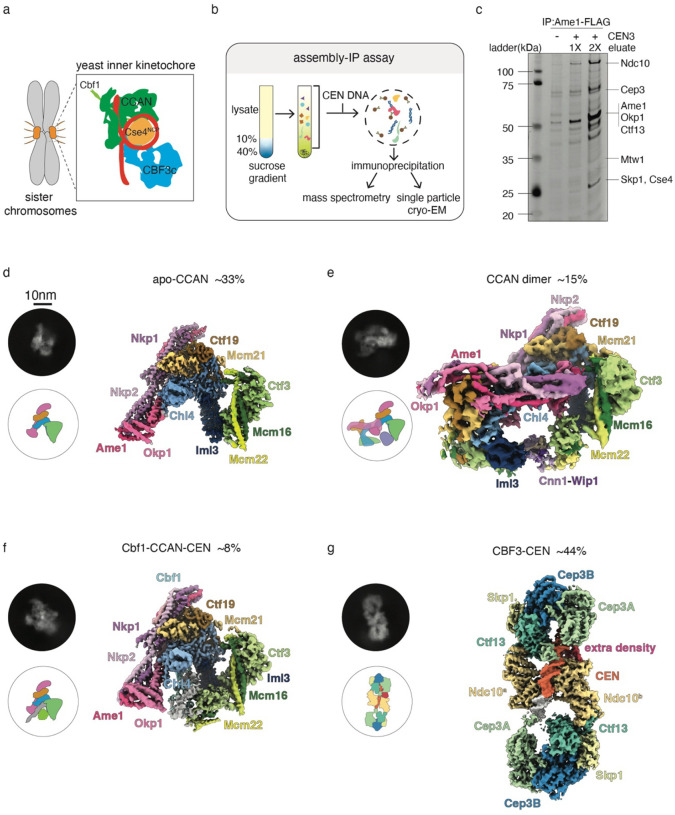
The purification of native kinetochore complexes and cryoEM overview of identified structures. a) A cartoon of a pair of budding yeast sister chromosomes with kinetochores colored in orange. The composition of the inner kinetochore complexes is illustrated in the box. CCAN is the constitutive centromere associated network, and NCP is the nucleosome core particle. b) Flowchart of assembly-IP method for native kinetochore complex purification. c) Silver-stained SDS-PAGE of Ame1-3XFLAG (SBY21782) IP or assembly-IP eluates. The presence of CEN3 DNA is indicated and the sample loading in lane 4 is twice amount of sample in lane 3. Kinetochore proteins are labeled based on the protein band molecular weight. d) The four structures solved from the assembly-IP assay. Representative 2D classifications and the density maps are shown for each structure. The name of the proteins are labeled in the corresponding color and the relative percentage of each complex detected is reported. The scale bar is 10 nm.

Centromeric chromatin serves as a platform to assemble the inner kinetochore, which is made up of a large constitutive centromere-associated network (CCAN)^6,7^ (Fig. 1a). The inner kinetochore serves as a bridge to the outer kinetochore that binds to microtubules and contains multiple copies of the Mis12, KNL1 and Ndc80 subcomplexes, as well as additional microtubule associated proteins such as the Ska1 complex or its budding yeast ortholog, the Dam1 complex^8–10^. Although kinetochore components are well defined, a complete structural understanding of how these proteins assemble in the native context to make functional kinetochores is lacking.

In *Saccharomyces cerevisiae*, the centromere is unique and sequence-specified by a ~125 bp sequence that assembles a single essential centromeric nucleosome required for kinetochore assembly^11,12^. The yeast centromere is divided into three centromere determining elements: CDEI, CDEII and CDEIII^3,13^. The CDEI and CDEIII elements are sequence defined^14^: CDEI is a palindromic, conserved DNA sequence that interacts specifically with a dimer of the transcription factor Cbf1^15,16^, while CDEIII is a 25 bp element containing several key nucleotides that are responsible for the recruitment of the CBF3 complex^17–19^. In contrast, CDEII is not sequence defined, but it is extremely AT-rich, with many homopolymer runs of A or T repeats spanning ~80 bp that are required for accurate centromere function^20^. However, A-T homopolymers are relatively stiff and are predicted to be poor templates for nucleosome formation^20^. Consistent with this, reconstitution experiments in which Cse4, the yeast CENP-A histone variant, were added to native CEN DNA result in limited and unstable nucleosome formation^21,22^. Thus, although the yeast centromere must form an essential centromeric nucleosome, it is paradoxically a very poor template for nucleosome formation. To address this problem, most inner kinetochore reconstitutions have replaced CDEII with a strong nucleosome positioning sequence or used a stabilizing antibody that blocks binding of the essential CCAN component Mif2 to assemble stable centromeric nucleosomes^21–24^. Together, these observations suggest that there are unidentified mechanisms that help bend the centromeric DNA and stabilize the centromeric nucleosome *in vivo*.

Yeast kinetochore assembly is initiated by the essential CBF3 complex (CBF3c) that enables deposition of the yeast centromeric histone variant, Cse4^2^. The CBF3core contains a Cep3 dimer, Ctf13, and Skp1. The Ndc10 protein loosely binds to the CBF3core to form the full CBF3c^25^. CBF3c interacts with the CDEIII element via a CCG motif that binds to the GAL4-like domain of Cep3^26–29^. Although it was proposed that CBF3c helps bend DNA to contribute to nucleosome formation^25,27,28^, it is not clear how CBF3c bends DNA. The behavior of Ndc10 at the centromere is also unclear. Ndc10 is localized to kinetochores throughout the cell cycle *in vivo*^30^, but structural studies have suggested that Ndc10 must disassociate from CBF3c after centromeric nucleosome formation^21,31^.

To better understand the mechanisms that promote centromeric nucleosome and inner kinetochore assembly, additional structural studies are required. Most prior studies have used recombinantly purified and reconstituted complexes to achieve high resolution structures of CBF3c^25,32^, the apo CCAN^23,33^, and the nucleosome bound state of CCAN when stabilized by an antibody^21^. While these structures provide functional insights, they provide little information regarding the dynamic motions and behaviors that kinetochores must undergo throughout the cell cycle, as well as the possible formation of intermediate assemblages. In addition, post-translational modifications that regulate the kinetochore are lacking in current structures and many structures use truncated proteins.

To address these issues, we developed a new method to assemble and efficiently enrich native budding yeast kinetochore complexes for cryoEM studies. We identified native structures that largely agree with previous work but contain previously unknown features. We observed significant DNA bending in the Cbf1-CCAN-CEN and CBF3-CEN complexes that would facilitate centromeric nucleosome formation. DNA bending requires an additional structural component, not previously detected, that we identify as an Ndc10 trimer. Consistent with a key role in promoting centromeric nucleosome assembly, mutants that abolish the Ndc10 trimer are lethal due to a lack of kinetochore assembly. Together, our work uncovers structural details that contribute to understanding how the yeast inner kinetochore assembles and lays the foundation for future studies to elucidate kinetochore function.

## Results

### Purified *de novo* assembled-IP kinetochores consist of many states

Kinetochore proteins are expressed at low levels and a single centromeric nucleosome assembles the entire yeast kinetochore, so native material for structural analyses is limited^9^. We previously isolated native kinetochore material from *S. cerevisiae* using a 3xFLAG epitope tag on the Dsn1 kinetochore protein^34^. While this method is useful for biochemical and biophysical assays^34–36^, cryoEM is not possible due to contaminating proteins and aggregates. In addition, the prep cannot be purified through gel filtration steps due to its large size and small quantity. Dsn1 purification is also limiting for inner kinetochore proteins^34^. To address these issues, we developed a new protocol to obtain native kinetochores that have fewer non-specific co-purifying proteins and a higher yield of the inner kinetochore. We arrested budding yeast cells in mitosis with microtubule destabilizing drug benomyl and generated cell lysates. We then pre-cleared the lysates via a sucrose gradient to decrease contaminating proteins (Supplementary Fig. 1a). We next immunoprecipitated (IP) three different inner kinetochore proteins: Ame1, Chl4 and Mif2, and found that an Ame1 IP best enriches for inner kinetochore proteins (Supplementary Fig. 1b).

To further recruit inner kinetochore complexes, we combined a well-established kinetochore assembly assay^37^ with the Ame1-3xFLAG immunoprecipitation. We added 500 bp centromeric DNA fragments containing 125 bp of CEN3 and additional flanking pericentromeric DNA on both ends into the clarified lysate and let kinetochores assemble for 90 minutes (Fig.1b). We then immunoprecipitated Ame1-3xFLAG and eluted the material from beads using FLAG peptide. We call this purification method an “assembly-IP”. When the assembly-IP was assayed by silver-stain analysis of SDS-PAGE and immunoblotting against kinetochore proteins (Fig. 1c and Supplementary Fig.1c), we observed enrichment of inner kinetochore proteins such as the CBF3 and Ame1-Okp1 complexes. To fully analyze the composition of the final assembly-IP product, we performed mass spectrometry. We found that most kinetochore proteins were present and the inner kinetochore proteins were detected with relatively high PSMs (peptide-spectrum matches) (Table S1). Taken together, these data showed that the assembly-IP method is an excellent method to obtain native kinetochore material containing the inner kinetochore.

We next analyzed both the Ame1-3xFLAG IP and the assembly-IP eluates by cryoEM (Supplementary Fig. 1d–g, Supplementary Fig. 2) and compared the corresponding structures. When centromeric DNA was added prior to the IP, we identified four kinetochore complexes at reasonable resolution (3.2 Å to 8 Å), suggesting they could be intermediates in the assembly process. We built models for all four complexes by using either previously solved related kinetochore subcomplex structures as the initial models^21,32,33^ or AlphaFold predictions^38^. In summary, we identified a single copy apo-CCAN alone (33% of the particles at 3.2 Å resolution, Fig. 1d), a new CCAN dimer structure in a different conformation compared to a prior reconstituted version (15% at 8 Å resolution, Fig. 1e), a Cbf1-CCAN-CEN complex (8% at 3.9Å resolution, Fig. 1f), and a CBF3-CEN complex with a significant extra structural density that wasn’t visualized from previous study (44% at 3.5Å resolution, Fig. 1g and Supplementary Fig. 2). It is noteworthy that even though we observed a 2D classification corresponding to a nucleosome complex (Supplementary Fig. 1d, red box), we could not generate a density map for that structure. This observation suggests that the nucleosome is not stable upon cryoEM sample preparation, consistent with previous studies^21,22^.

### *De novo* assembled structures adopt unique conformations

Although the *de novo* assembled structures we identified are similar to previously reconstituted ones, there were some differences. The apo-CCAN complex (Fig. 1d) was the only kinetochore complex that we detected when we immunoprecipitated Ame1-3xFLAG directly in the absence of the assembly assay^37^ (Supplementary Fig. 1c). In agreement with a previously solved reconstituted CCAN structure^23,33^, the native CCAN complex adopts an “arrowhead” shape with the Ame1-Okp1 complex and the Nkp1-Nkp2 proteins forming one arm and the Iml3-Mcm16-Mcm22-Ctf3 proteins forming another arm. The two arms are bridged by the Chl4-Mcm21-Ctf19 proteins. When comparing the native and reconstituted structures, we found a slightly wider channel in the native structure when the Okp1-Ame1 arms are aligned (Supplementary Fig. 3a).

When CEN DNA was present prior to the purification, we detected three additional structures. One corresponds to Cbf1-CCAN-CEN. Even though we can’t distinguish DNA base pairs at 3.9 Å resolution, it is known that the Cbf1 dimer recognizes CACGTG in CDEI^15,16^. We therefore fit the model of a Cbf1 dimer bound to the CDEI sequence based on the previous crosslinking data and reconstituted structures^15,21,39^. In the Cbf1-CCAN-CEN structure we obtained, we observed a more dramatic bend in the CEN DNA (Supplementary Fig. 3b–c). Specifically, the CEN DNA is bent toward Cnn1-Wip1 by about 15° starting at CDEII (Supplementary Fig. 3b–e). This structural variation is likely due to CDEII sequence differences because the prior study replaced CDEII to reduce the high AT content and stabilize the reconstituted nucleosome^21^. Consistent with this, previous native polyacrylamide gel electrophoresis analyses on the budding yeast centromere confirmed a strong intrinsic curvature caused by the AT-rich CDEII^40^.

We also detected a unique CCAN dimer structure (Fig. 1e). There is a dramatic shift in the Okp1-Ame1 arm of the native CCAN dimer relative to previously reported reconstituted structures^23,33^, resulting in loose packing between the two CCANs (Supplementary Fig. 4a, right). In addition, there was additional density representing a flexible loop that exists between the two CCANs (Supplementary Fig. 4b). This density connects the foot of Okp1 from CCAN^a^ to Chl4 from CCAN^b^. Prior cross-linking mass spectrometry suggested a potential interaction^44^ between Mif2^343−363^ and Okp1^132–152^. In addition, an interaction between Mif2^256−388^ with the Chl4-Iml3 complex has been reported^41^. These data suggest that the unknown density in our structure might be Mif2^343–363^. Interestingly, we only detected the CCAN dimer in the assembly-IP material but not in the direct Ame1-3XFLAG IP (Supplementary Fig. 1d), suggesting that CEN DNA is required for CCAN dimer formation. Consistent with this, the percentage of CCAN dimer particles increased as more DNA was added to the assembly assays (Supplementary Fig. 4c), strongly suggesting that *de novo* kinetochore assembly was required to detect the CCAN dimer and that this may be an intermediate assembly step.

### The native CBF3 complexes interact with CEN DNA tightly and induce a sharp bend

**Figure 2.**
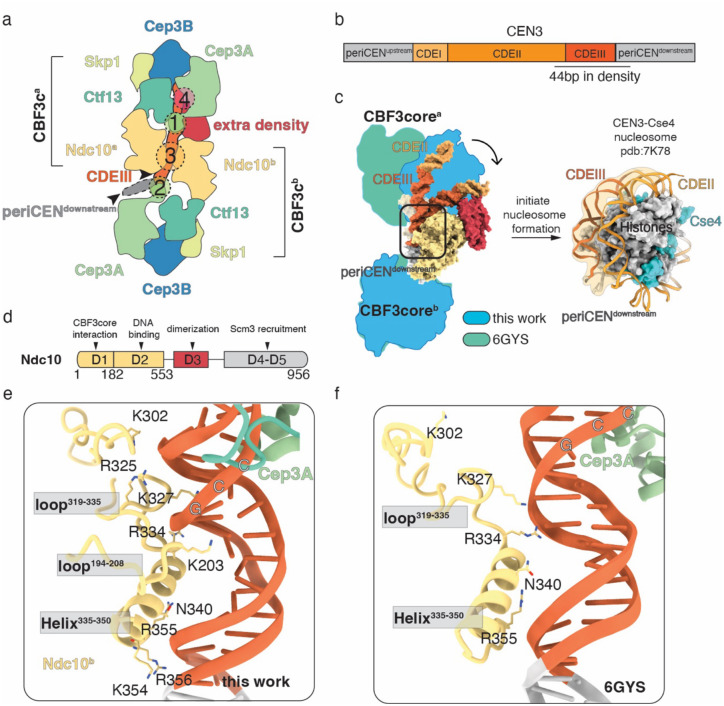
Comparison of native and recombinant reconstituted CBF3-CEN structures. a). Cartoon of the organization of the CBF3-CEN complex. Each CBF3c protein is colored and labeled. The arrowheads point to CDEIII that tightly interacts with CBF3c and periCEN^downstream^. The four protein-DNA interaction surfaces are outlined by dashed circles and labeled with numbers. b). Color scheme of CEN3 used in assembly assay. Based on our structure, 44 bp DNA protected by CBF3 complexes are underlined. The periCENs are colored in grey. CDEI is colored in light orange. CDEII is colored in orange. CDEIII is colored in dark orange. c). Illustration of CEN DNA bending by superimposing the recombinant reconstituted CBF3-CEN^32^ (PDB: 6GYS) and native CBF3-CEN structures on CBF3core^b^. The movement of DNA is indicated by the arrow. The extra density is colored in bright red. The interaction surface between Ndc10^b^ and CEN DNA is boxed with a black rectangle, and the details are highlighted in (e.) and (f.). To illustrate that CEN DNA bending corresponds to the angle in a Cse4 nucleosome, a previously solved Cse4 nucleosome (PDB:7k78) structure^22^ is shown. The CEN DNA is colored in the same scheme as on the left. Cse4 is colored in cyan. PeriCEN, CDEIII and the small part of CDEII are in transparent shade to compare the bending angle with the CEN DNA on the left. d). Cartoon of Ndc10 domain organization. D1-D2 are labeled and colored in yellow, which is well-structured and resolved in our structure. D3 is colored in bright red, and its structure is solved in this work. D4-D5 are not resolvable and colored in grey. The proposed function of each domain is labeled. e-f).Interaction between Ndc10^b^ and CEN DNA in the native CBF3-CEN structure (e) and the recombinant reconstituted structure ((f),PDB: 6GYS) shows that there are more Ndc10^b^ residues in the native structure that contact the CEN DNA. Ndc10^b^ is colored in yellow, the CDEIII DNA is colored in orange and GAL4 from Cep3A is colored in light green. The CCG motif is highlighted.

We identified a CBF3c dimer that contained a previously undetected extra density, interacting with CEN DNA. Using AlphaFold 3 prediction and referring to a previously published reconstituted CBF3-CEN structure^32^, we were able to generate a native CBF3-CEN complex model. The CEN DNA threaded between the two CBF3 complexes and contacted it in four places (Fig. 2a–b). As previously reported^28,31,32,42^, Cep3A from CBF3c^a^ binds to the conserved CCG motif in CDEIII and Cep3A from CBF3c^b^ binds to periCEN through a non-sequence specific interaction (Fig. 2a, interactions 1 and 2 and Supplementary Fig. 5a–b). Ndc10 proteins from each CBF3c clamp onto CDEIII and the periCEN through non-specific DNA interactions^28,32^ (Fig. 2a, interaction 3). Notably, we also observed that the extra density interacts with CDEII adjacent to the CDEIII element (Fig. 2a, interaction 4).

To compare the native and reconstituted structures, we superimposed the two CBF3core^b^ complexes from each structure. When they are aligned, we found that the CBF3c^a^ complexes bind to CEN DNA differently, which results in a large bend and kink in the CDEIII element close to the periCEN^downstream^ in the structure we identified (Fig. 2c, left, and movie 1). The bend in the structure we identified matches the angle in the nucleosome structure (Fig. 2c, right), suggesting that CBF3 binding may facilitate the initiation of centromeric nucleosome assembly by bending the centromeric DNA.

The Ndc10 polypeptide sequence is roughly divided into five domains with D2 mediating DNA binding^43^ (Fig. 2d). We detected more Ndc10 residues interacting with CEN DNA in the native structure (Fig. 2e) compared to the reconstituted one (Fig. 2f). Consistent with prior work, we found that D2 domain residues in Ndc10^b^ insert into the major groove immediately downstream of the CDEIII motif that interacts with Cep3A. This reinforces the Cep3A interaction with the CEN DNA and positions Ndc10 closer to the major groove (Fig. 2e). We also detected additional CEN DNA contact points through two lysines from Ndc10^b^ loop^319−335^ and another Ndc10^b^ loop^194−208^ that interacts with the DNA sugar backbone (Fig. 2e–f). Ndc10^a^, the other copy of Ndc10, interacts with the periCEN^downstream^ and 3’ end of CDEIII through the same residues. Because we observed strong extra density that interacts with the junction between CDEIII and CDEII, we propose that this interaction causes an initial bending of the centromeric DNA at CDEIII, which allosterically induces the additional interactions to reinforce the stability of the curved DNA.

### Identification of an Ndc10 trimer domain whose interaction with Ndc10^D1_D2^ is important for kinetochore assembly

The extra structural density that closely interacts with Ndc10^b^ and several base pairs at the end of CDEII is very strong and present in all of our CBF3-CEN 3D classifications and even in the structure of the monomeric CBF3 core and CEN DNA, indicating a strong interaction between the extra density and Ndc10, the core CBF3 complex and the centromeric DNA (Supplementary Fig. 5c, class 6). In addition, even though the CBF3-CEN structure is symmetrical in the native structure, only CBF3^a^ interacts with the extra density and the other side of the CBF3 complex lacks this strong extra density. Taken together, these data suggest that the extra density represents an early step in inner kinetochore assembly that may have an important functional role, such as helping to bend the CEN DNA and stabilize the centromeric nucleosome.

**Figure 3.**
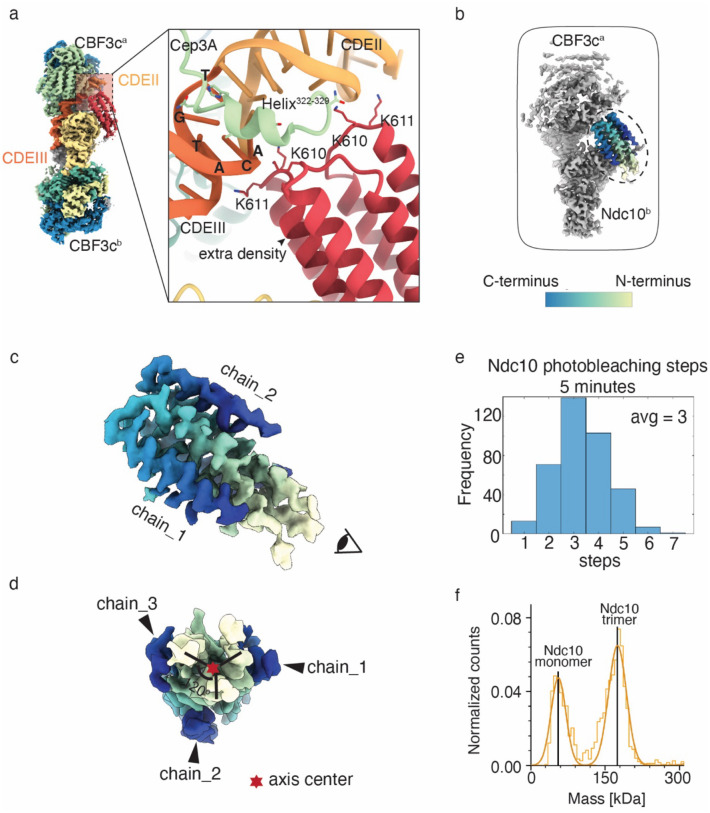
The extra structural density in the native CBF3-CEN complex is a trimer of Ndc10579–639. a). Detailed interaction between the extra density and the CEN DNA. The model of the extra density is colored in bright red. CDEIII is in dark orange and CDEII is in orange. b). Focus refined density map shows well resolved extra density in the box. The extra density is colored and circled with a dashed line. The C-terminus of the helix-loop-helix is colored in blue, and the N-terminus of the helix-loop-helix is colored in pale yellow. c). Zoom-in view of the extra density adopting the same view from b. d). Zoom-in view of the extra density observed from the cartoon eye angle in (c). Each of the chains is labeled and the symmetry axis is marked by a red star. The C-terminus and N-terminus are indicated by blue and pale yellow, respectively. e). The frequency of photobleaching steps on kinetochores assembled from extracts from Ndc10-GFP cells (SBY22191) via a single molecule TIRF assay after *de novo* assembly for 5 minutes. f). Mass photometry of Ndc10^D3-D5^ purified from *E.coli*. Two major peaks were observed and correspond to an Ndc10^D3-D5^ monomer and trimer, respectively.

To identify the extra density, we performed local 3D classification by masking the particles that have the strongest extra density in cryoSPARC^44^ and then performing local refinement with the extra density center as the fulcrum. This improved the local resolution and density connectivity, allowing the side chains to become clear (Supplementary Fig. 6a-b). We then predicted its sequence through model-angelo^45^ and ran it through a blast prediction program. The extra density was predicted to correspond to three chains of helix-loop-helix (HLH) hairpin structures that all have about 30% sequence identity with Ndc10 residues 579–639 (Supplementary Fig. 6c). When we modeled the extra density with the Ndc10^579−639^ sequence, the overall fit was good, particularly for some bulky side chains of phenylalanine and tryptophan (Supplementary Fig. 6b). Two pairs of 610K and 611K from two Ndc10^579−634^ regions were involved in DNA binding (Fig. 3a).

It was previously proposed that the *K. lactis* Ndc10 forms dimers through D3^43^. We find that Ndc10^579−634^, which is in D3, is responsible for Ndc10 oligomerization so we have named it Ndc10^D3_trimer^. We observe strict threefold symmetry of Ndc10^D3_trimer^ density (Fig. 3b–d). To validate that Ndc10 forms a trimer, we used a TIRF microscopy assay to analyze the Ndc10 copy number on assembled kinetochores via photobleaching^46,47^. In this assay, fluorescently labeled CEN DNA molecules are tethered to a coverslip and incubated with lysate made from cells expressing Ndc10-GFP. Within 5 minutes of assembly, there were approximately 3 steps of Ndc10-GFP photobleaching for 40% of the CEN DNA molecules (Fig. 3e). Our prior work indicated that the inner kinetochore takes more than 30 minutes to assemble in this assay^46^, suggesting that the Ndc10 trimer forms in the absence of the rest of the inner kinetochore. The number stayed relatively similar after 30 minutes of assembly (Supplementary Fig. 6d), indicating that three Ndc10 molecules are maintained as the inner kinetochore is assembled.

To further validate Ndc10 trimerization, we purified a recombinant Ndc10 fragment spanning residues 541 to 956 (D3-D5) from bacteria (~52kDa). We analyzed the molecular weight by mass photometry and found two major peaks corresponding to a monomer (~53kD) and a trimer (~150kD) (Fig. 3f). We utilized Alphafold 3^38^ to predict the structure of Ndc10^D3-D5^ (Supplementary Fig. 6e, left). When zooming in the Ndc10^579−634^ region, we observed the same trimer structure that we identified in cryoEM with high confidence (Supplementary Fig. 6e, right).

**Figure 4.**
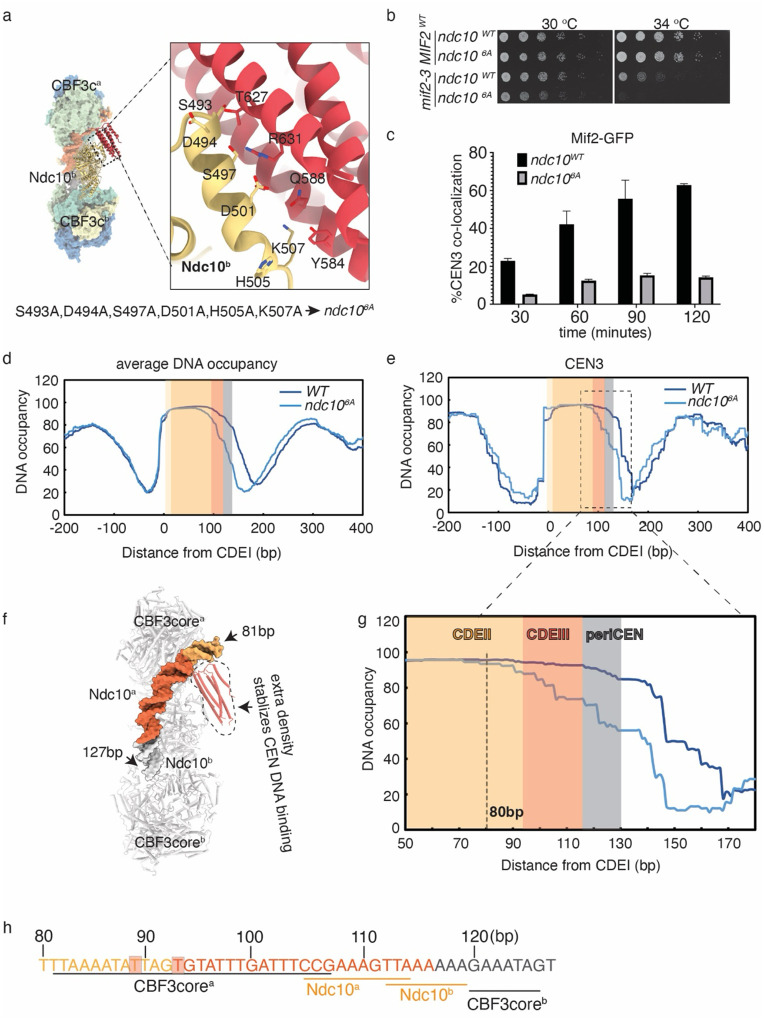
The interaction between Ndc10^D3_trimer^ and Ndc10^D1-D2^ from Ndc10^b^ stabilizes the association of the kinetochore and centromere. a). Residues involved in the interactions between Ndc10^D3_trimer^ and Ndc10^b^. Ndc10^b^ is yellow and the Ndc10^D3_trimer^ is bright red. The residues that are mutated in *ndc10*^*6A*^ are labeled. b). Five-fold serial dilutions of WT (SBY3), *ndc10*^*6A*^ (SBY23670), *mif2-3* (SBY21479) and *ndc10*^*6A*^ *mif2-3* (SBY23913) cells were plated on 2% glucose at 30 °C and 34 °C. c). Co-localization of Mif2-GFP and CEN3 DNA measured by a single molecule TIRF assay using *MIF2-GFP* (SBY22094) and *MIF2-GFP ndc10*^*6A*^ (SBY24238) strains. d). Average DNA occupancy of all centromeres in WT (SBY3) or *ndc10*^*6A*^ (SBY23670) yeast strains. WT or *ndc10*^*6A*^ strains were traced with dark blue or light blue lines, respectively. The beginning of CDEI is assigned as 0 bp. CDEI is pale yellow, CDEII is orange, CDEIII is dark orange and the periCEN is grey. e). DNA occupancy of CEN3 and its neighboring DNA in WT or *ndc10*^*6A*^ strains were traced with dark blue or light blue lines, respectively. The beginning of CDEI is assigned as 0 bp. CDEI is pale orange, CDEII is orange, CDEIII is dark orange and the periCEN is grey. f). Surface showing relative direction of CEN DNA bound to CBF3. g). Zoom-in view from f. The significant drop of DNA occupancy starting in CDEII 80 bp from CDEIII is highlighted. h). The sequence of CEN DNA that is protected by the CBF3-CEN complex is illustrated with CDEII in light orange, CDEIII in dark orange and the periCEN in grey. The regions of CEN DNA that interact with components of the CBF3 complex are labeled. The two bases that are boxed in a pink shade interact with extra density.

From the model we built earlier, we identified six residues within the D1/D2 domain of Ndc10 that contact the extra density now recognized to be its D3-trimer domain: S493, D494, S497, D501, H505 and K507 (Fig. 4a). To determine whether the interaction between the Ndc10^D3_trimer^ and Ndc10^b (D1-D2)^ affects cellular fitness, we mutated these residues to alanine (*ndc10*^*6A*^). The *ndc10*^*6A*^ mutant did not have any obvious growth defects, so we crossed it to a temperature sensitive *mif2-*3 mutant to determine if it exhibits genetic interactions with another inner kinetochore mutant. At a semi-permissive temperature, the double mutant was lethal, indicating that the Ndc10^D3_trimer^ density contributes to inner kinetochore function (Fig. 4b). To further explore the effects of the *ndc10*^*6A*^ mutant, we performed a *de novo* kinetochore assembly assay^37^. We found that both Ndc10^WT^ and Ndc10^6A^ assembled onto CEN DNA normally (Supplementary Fig. 6f). However, Mif2 and Okp1 assembly were strongly decreased, and Cse4 recruitment was slightly impaired, suggesting that inner kinetochore assembly is compromised in *ndc10*^*6A*^ cells (Supplementary Fig. 6e). To monitor kinetochore assembly efficiency at the single molecule level, we used the TIRF assembly assay to assay Mif2-GFP yeast extract on slides containing fluorescently marked CEN3. Like the bulk assembly assay, Mif2 co-localization with CEN3 DNA was reduced in the *ndc10*^*6A*^ lysate (Figure 4c). Taken together, these indicate that disrupting the binding between the Ndc10^D3_trimer^ and Ndc10^D1-D2^ from CBF3c^2^ compromises inner kinetochore assembly efficiency.

The presence of the Ndc10^D3_trimer^ led to a stronger DNA density than previously detected in a reconstituted structure of CBF3core with an Ndc10-D1-D2 fragment^32^, suggesting that the Ndc10^D3_trimer^ stabilized the interaction between CBF3 complexes and DNA. To test this, we analyzed DNA occupancy at endogenous centromeres in WT and *ndc10*^*6A*^ cells using single molecule chromatin fiber sequencing (Fiber-seq)^48^. Spheroplasted cells were exposed to an N6-adenine DNA methyltransferase (m6AMTase) that nonspecifically methylates accessible regions of DNA. The resulting m6A-labeled chromatin fibers were then analyzed using long-read single-molecule sequencing at greater than 100× genomic coverage, allowing the detection of individual protein-binding events with nearly single-nucleotide resolution at all 16 centromeres.

Using computational tools, we calculated the percentage of DNA occupancy at each bp surrounding each centromere. Roughly 160 bp DNA is protected at centromeres in wild-type cells, encompassing ~10 bp upstream of the CDEI element, the entire ~120 bp of CEN DNA, and ~30 bp downstream of the CDEIII element, corresponding to the binding of Cbf1, the Cse4 nucleosome and the CBF3 complex as previously reported^49^ (Fig. 4d and 4e, dark blue line). Strikingly, the nucleosome footprint at centromeres was shorter in the *ndc10*^*6A*^ cells, occupying less DNA predominantly on the CDEIII and CDEIII-adjacent sides (Fig. 4d and 4e, light blue line). At individual centromeres, like CEN3, we detected a reduction in DNA occupancy starting around 80 bp – towards the end of the CDEII element – which overlaps with the tight CBF3 complexes binding to DNA observed in our native structure (Fig. 4f–h). Overall, these data suggest that the interaction between the Ndc10^D3_trimer^ and Ndc10^D1-D2^ we identified stabilizes CBF3c on DNA *in vivo*.

### Mutating the Ndc10^D3_trimer^ self-interaction surface abolished Ndc10 function *in vivo*

**Figure 5.**
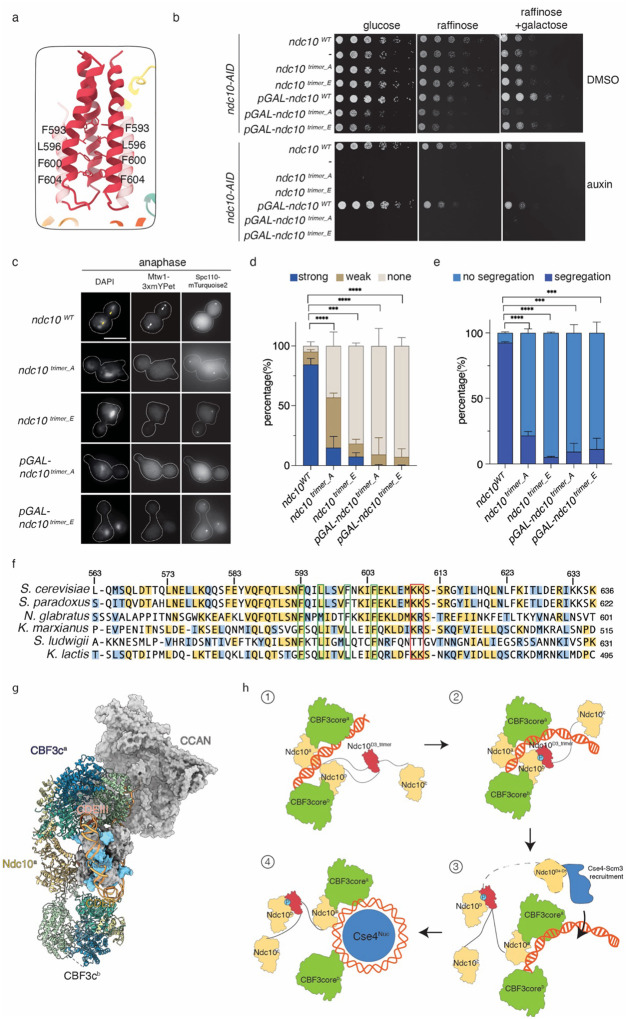
Ndc10 trimerization is essential for kinetochore assembly and cell viability. a). Zoom-in model shows residues involved in the Ndc10 trimerization. b). Five-fold serial dilutions of WT (SBY12796), *ndc10-AID* (SBY21327), *ndc10-AID ndc10*^*trimer_A*^ (SBY24533), *ndc10-AID ndc10* ^*trimer_E*^ (SBY24478), *ndc10-AID pGAL-ndc10*^*WT*^ (SBY24690), *ndc10-AID pGAL-ndc10*^*trimer_A*^ (SBY24689), *ndc10-AID pGAL-ndc10*^*trimer_E*^ (SBY24687) strains were grown on either 2% glucose, 2% raffinose or 2% raffinose and galactose. 500 uM auxin was used to degrade the Ndc10-AID protein. c). Mtw1-3xmYPet and Spc110-mTurquoise2 label the kinetochores and spindle pole bodies in WT (SBY24595), *ndc10-AID, ndc10*^*trimer_E*^ (SBY24691), *ndc10-AID, ndc10*^*trimer_A*^ (SBY24692), *ndc10-AID, pGAL-ndc10*^*trimer_E*^ (SBY24687), *and ndc10-AID, pGAL-ndc10*^*trimer_A*^ (SBY24689) cells. The white arrowheads indicate strong kinetochore foci which were missing in mutant cells. Yellow arrowheads indicate chromosomes that segregated into two daughter cells. Representative pictures are shown for anaphase cells that were determined based on spindle pole body positions. The scale bar is 5 um. d). Quantification of kinetochore signal strength by measuring the Mtw1-3xmYPet in all cell cycle stages. All the images were normalized with the same threshold and the foci that are equally strong to WT Ndc10 are listed as strong while faint Mtw1 foci are listed as weak. When no foci were observed in a cell, it is listed as none. 200 cells were counted for each replicate, and three biological replicates were used to measure the significance through two-tailed t test. **** P<0.00001, *** P< 0.0001. e). Quantification of chromosome segregation by observing DAPI signals. Only anaphase cells are counted, marked by separated Spc110-mTurquoise2 in daughter cells. When an anaphase cell has DAPI signals evenly distributed into the daughter cells, it is listed as segregation. When an anaphase cell has only one DAPI signal, it is listed as no segregation. ~60 cells were counted for each replicate, and three biological replicates were used to measure the significance through two-tailed t test. **** P<0.00001, *** P< 0.0001. f). Sequence alignment of Ndc10^D3^ through many fungal species. The residues colored in yellow are highly conserved and the residues colored in blue are medium conservation. The DNA binding residues are highlighted with a red box and the residues that are critical for trimer formation are colored in a green box. All trimer residues are conserved throughout the alignment. g). Proposed model of one structure involved in kinetochore assembly initiation. The model of CBF3 complex and CCAN were solve from this work. The nucleosome was used from PDB: 8ow0. h). Proposed intermediate steps of kinetochore assembly. The CBF3core is colored in green. The Ndc10^D1-D2^ is colored in yellow. The unstructured region between D2 and D3 is illustrated with curved lines. Potential phosphorylation is colored in blue with letter P. In ③, Ndc10^D4-D5^ is connected through a dashed line to Ndc10^D3_trimer^ and interacts with Cse4-Scm3 complex to guide its deposition to centromere. Details of model are discussed in text.

The three chains of the Ndc10^D3_trimer^ interact through hydrophobic interactions utilizing F593, F596, F600 and F604 (Fig. 5a). This hydrophobic patch includes all three α-helices compactly packed. To test whether this interaction is important, we mutated the four residues to alanine or aspartic acid (*ndc10* ^*trimer_A*^ or *ndc10* ^*trimer_E*^) to abolish the trimer. We then purified the Ndc10^D3-D5^ fragment with *ndc10* ^*trimer_A*^ or *ndc10* ^*trimer_E*^ (Supplementary Fig. 7a) and measured their molecular weight using mass photometry. As expected, the 150 kDa trimer peak was not observed and there was a single major peak at ~55 kDa from both mutant proteins indicating the trimer was abolished (Supplementary Fig. 7b). We next expressed the mutants in a strain where the endogenous Ndc10 protein was fused to an auxin-inducible tag to control its degradation (*ndc10-AID*)^50^. As expected, the *ndc10-AID* strain dies on auxin and is rescued by a wild-type copy of *NDC10* (Fig. 5b). Strikingly, the two *ndc10* mutants were inviable on auxin, indicating that the hydrophobic interaction within Ndc10 is essential (Fig. 5b).

To determine whether the mutants affect kinetochore assembly, we performed a bulk kinetochore assembly assay after auxin addition to the strains^37^. We noticed that the steady state levels of the Ndc10 mutants were lower than WT Ndc10 (Supplementary Fig. 7c). We therefore expressed the Ndc10 mutants from the galactose inducible promoter to restore their protein levels prior to performing the assembly assay (Supplementary Fig. 7c). There was a defect in kinetochore assembly in the mutant lysates (Supplementary Fig. 7d, lanes 4 and 6). Although the overexpressed Ndc10 proteins assembled onto the centromeric DNA at normal levels, there was a strong defect in inner kinetochore assembly and the cells remained inviable (Supplementary Fig. 7d, lanes 4 and 6, Fig. 5b).

To determine the *in vivo* consequences, we expressed the *ndc10* mutants in an *ndc10-AID* strain with fluorescently marked kinetochores (Mtw1-3xmYPet)^51^ and spindle pole bodies (Spc110-mturquoise2)^52^. We synchronized the cells in G1 phase with α-factor in the presence of auxin and then released them into the cell cycle in auxin. After 105 mins, most cells had entered anaphase as defined by the separation of the two spindle pole bodies into each daughter cell (Fig. 5c). In WT cells, the kinetochore signals were strong and co-localized near the spindle pole body in both metaphase and anaphase cells. However, in *ndc10* ^*trimer_A*^ mutant cells, the Mtw1 signal was extremely weak. The phenotype was even more striking in the *ndc10* ^*trimer_E*^ mutant strain where most cells completely lost Mtw1 signal (Fig. 5c–d). We assayed chromosome segregation by staining the cells with DAPI and quantifying the cells whose spindle pole bodies separated to daughter cells. In the WT strain, more than 90% of cells equally separated the chromosomal DNA. We presume that the mild segregation defect in the control strain is due to having epitope tags on multiple kinetochore proteins. In contrast, the *ndc10* mutants exhibited severe chromosome segregation defects, and the DNA remained in the mother cell most of the time (Fig. 5c–e). The lack of kinetochore assembly explains why the cells do not arrest in metaphase via the spindle assembly checkpoint and are able to elongate their spindle but fail to segregate chromosomes. Taken together, these data indicate that Ndc10 trimerization has an essential role in kinetochore assembly and chromosome segregation.

## Discussion

We developed an efficient system to purify native kinetochore complexes from budding yeast for cryoEM structural studies. This method overcomes the difficulty of purifying native kinetochore complexes due to their extremely low expression levels and maintains post-translational modifications and native CEN DNA that are absent in prior reconstitution studies. By assembling *de novo* kinetochores with native components, we were able to identify inner kinetochore complexes that had not been previously detected and speculate that some of the structures may be intermediate steps in kinetochore assembly. However, we did not detect outer kinetochore complexes. We presume this can be addressed in the future by performing an IP against outer kinetochore proteins after *de novo* assembly.

Although CEN DNA was essential for *de novo* kinetochore assembly, we did not detect any DNA density in our apo-CCAN or CCAN dimer density maps. We also did not visualize the largely unstructured Mif2 and Scm3 proteins that interact with both CCAN and DNA^53,54^. The essentiality of CEN DNA for *de novo* assembly, together with the abundance of Mif2 and presence of Scm3 in the assembly IPs (Table S1) suggest that the CEN DNA is present but not resolvable in these structures because it is bound via these largely unstructured proteins, Mif2 and/or Scm3. We also did not detect the Cse4 nucleosome in complex with any of the inner kinetochore structures despite confirming its presence in the assembly-IP by immunoblotting and mass spectrometry. Therefore, even in the presence of native kinetochore subcomplexes, the Cse4 nucleosome is not stable enough to be solved at high resolution. One possible explanation is that the kinetochore complexes containing nucleosomes were not abundant enough to obtain an averaged density map, possibly because they are more intrinsically dynamic. In the future, this difficulty might be overcome by enriching assembled kinetochores on grids coated with antibody^55^ or covalently decorating CEN DNA^56^ on the grid to perform assembly assay on grids.

Our assembly-IP protocol enriched kinetochore proteins via the CCAN component Ame1. However, the CBF3-CEN complex we purified isn’t known to directly associate with Ame1. One explanation is that the larger CBF3-CEN-CCAN complex collapsed during vitrification. To address this, we carefully analyzed negative stain datasets of Ame1-3XFLAG assembly-IP material by averaging particles into larger 2D classes. However, we still could not visualize any average that resembled a complex of CBF3-CEN and CCAN together. Another possibility is that the two complexes were linked through CEN DNA. Because the Cse4 nucleosome is not stable, we assume a large region of CEN DNA isn’t wrapped in a well-defined structure. In this case, we can only solve kinetochore complex structures containing CEN DNA at either the CDEI end or CDEIII end. In the future, studies using other inner kinetochore proteins for the assembly-IP may help resolve additional structures.

We identified a previously unknown structure of Ndc10^D3^ that is essential for kinetochore assembly and cell viability. We found that the Ndc10 D3 domain proposed to mediate dimerization^43^ actually forms a trimer. Consistent with this, sequence alignment of multiple fungal species shows that the hydrophobic residues responsible for Ndc10 trimerization are highly conserved (Fig. 5f). Our findings agree with fluorescent tagging studies of Ndc10 that quantified *in vivo* copy number^57^. Although Ndc10 is a kinetochore protein throughout the cell cycle, it also localizes to the spindle and bud neck after anaphase onset^30^. One possibility is that the third Ndc10 that bundles with the two CBF3-bound Ndc10^D3^ can extend to interact with spindle microtubules^30^. Our work is consistent with an earlier study that proposed an extended multimeric Ndc10 structure over the centromere^29^. Ndc10^D3^ also interacts with CEN DNA, and it will be important to understand if this interaction is sequence specific and the consequences of abolishing it in the future.

We also found that the Ndc10 trimer helps to stabilize CBF3 with DNA and that the native CEN sequences exhibit more bending than prior structures. Because the yeast centromere is a poor template for nucleosome assembly, the properties we identified may facilitate its bending to form the centromeric nucleosome. It is unclear why Ndc10 trimerization was not previously visualized in reconstituted structures, but some of the differences between structures we found may be due to post-translational modifications. We observed several phosphorylation events on Ndc10 when we analyzed the assembly-IP material by mass spectrometry, and it will be important to study their function in the future.

Because we used native CEN DNA, our work identified two previously undetected sharp DNA bends: one between the junctions of CDEII and CDEIII and the other between CDEI and CDEII. If we fit a centromeric nucleosome (PDB: 8ow0) into a structure that maintains these bends, it cannot accommodate two CCAN, two CBF3 complexes and an Ndc10 trimer domain. However, we were able to fit two CBF3 core complexes, one CCAN and one Ndc10 association without steric clashes (Fig, 5g). Based on this hypothesized structure, we propose the following initial steps of kinetochore assembly. First, two complete CBF3 complexes will be deposited on CDEIII via sequence specific recognition (Fig. 5h ①). There are three Ndc10s involved in this interaction. Two Ndc10s are incorporated into each of the CBF3 complexes, while a third Ndc10 loosely interacts through the Ndc10^D3_trimer^. The Ndc10^D3_trimer^ then associates with Ndc10^b^ to complete the bending of CDEIII (Fig. 5h ②). Scm3-Cse4 are recruited to Ndc10^D4-D5^ to initiate Cse4 nucleosome formation^43^ (Fig. 5h ③). The rest of the histones and inner kinetochore CCAN proteins are then recruited to help stabilize the Cse4 nucleosome and Scm3 is released (Fig 5h ④).

At this point, the structure cannot accommodate two of the Ndc10 proteins, so we propose that Ndc10^b^ and the Ndc10^D3_trimer^ remain connected but dissociate from the CBF3core^b^, consistent with a previous publication stating that the interaction between Ndc10 and CBF3core is weak^25^. One possibility is that this allows the two Ndc10s that are not associated with any inner kinetochore proteins to contact microtubules. Although we and others have detected a CCAN dimer, we note that disrupting the interaction between the Ndc10^D3_trimer^ and the Ndc10^D1-D2^ proteins in the Ndc10^6A^ mutant protein abolished protection of CDEIII in the Fiber-seq assay. This suggests that the CDEIII protection is mediated by CBF3 as opposed to a second copy of CCAN, or that this mutant specifically disrupts one CCAN copy.

In summary, we purified and identified native yeast high resolution kinetochore structures for the first time and found complex conformations that were not previously detected in reconstitutions using recombinantly purified proteins. This work allowed us to elucidate a previously unknown Ndc10 trimerization that is conserved in sequence and essential for kinetochore assembly and chromosome segregation. Our work supports the previous proposal that Ndc10 is a central scaffold for inner kinetochore assembly^43^. Because other DNA-protein complexes are regulated by multiple steps and are highly dynamic, such as chromatin remodelers and RNA transcription complexes, structural studies on these complexes may also benefit from using natively assembled complexes in the future.

## Materials and Methods

### Yeast strains

Media and genetic and microbial techniques were essentially as described^58^. Yeast strains used in this study are listed in Table S2. The 6His–3XFLAG epitope tagging of the endogenous *AME1, Mif2* and *CHL4* genes was performed using a PCR-based integration system using primers SB2434-SB2435 and plasmid pSB1590 as a template. All tagged strains we constructed are functional *in vivo* and do not cause any detectable growth defects or temperature sensitivity.

### Growth and lysate preparations from budding yeast

All yeast growth was performed as described previously^34^. Briefly, yeast were grown in YPD (1% yeast extract, 2% peptone, 2% D-glucose). Large cultures of SBY21782 (*AME1–3XFLAG*) were grown on shakers (220 rpm) at 23 °C. Cultures were treated with benomyl at a final concentration of 30 μg/ml (1:1 addition of 60 μg/ml benomyl YEP media) for 2 hours at 23 °C and then harvested by centrifugation for 10 minutes at 5000xg at 4 °C. Kinetochore material was purified based on a previously described protocol^34,37^. Briefly, the endogenous *AME1* kinetochore gene was C-terminally tagged with 6xHis and 3xFLAG. Harvested yeast were resuspended in Buffer H/0.15 (25 mM HEPES pH 8.0, 150 mM KCl, 2 mM MgCl_2_, 0.1 mM EDTA pH 8.0, 0.1% NP-40, 15% glycerol) supplemented with protease inhibitors, phosphatase inhibitors, and 2 mM DTT. After resuspension and re-spinning, yeast pellets were frozen in liquid nitrogen and lysed using a freezer mill (SPEX, Metuchen NJ). Lysates were first treated with 50 U/mL benzonase (Sigma-Aldrich) at room temperature for 30 minutes. The treated lysate was then clarified via tabletop centrifugation at 16,000g for 30 minutes and the supernatant was pooled. The sucrose gradient was generated by layering 1.5 mL of 40%, 25% and 10% sucrose in Buffer H/0.15 with no glycerol (25 mM HEPES pH 8.0, 150 mM KCl, 2 mM MgCl_2_, 0.1 mM EDTA pH 8.0, 0.1% NP-40) supplemented with protease inhibitors, phosphatase inhibitors and 2 mM DTT. The pooled supernatant was loaded on sucrose gradient. The lysate was further clarified via ultracentrifugation at 27,000 rpm for 16 hours, allowing contaminant proteins to pellet. The clarified layer was extracted with syringe. The extract was snap frozen with liquid nitrogen and stored in −80 °C until use.

### Purification of kinetochore complexes

For Ame1-3XFLAG IP, extracts prepared as described above were incubated with magnetic α-FLAG antibody conjugated Dynabeads (Invitrogen, Waltham MA) for 90 minutes at 4 °C with rotation. For Ame1-3XFLAG assembly IP, 18,750 ng sonicated single strand salmon sperm DNA (ssssDNA) per ml lysate was incubated with the extract on ice for 15 minutes and 625 ng 500 bp CEN DNA per ml lysate was rotated at 23 °C for 90 mins before adding magnetic α-FLAG antibody conjugated Dynabeads. The sequence of 500bp CEN DNA is: 5’ATCAGCGCCAAACAATATGGAAAATCCACAGAAAGCTATTCATTGAAAAAATAGTACAAATAAGTCACATGATGATATTTGATTTTATTATATTTTTAAAAAAAGTAAAAAATAAAAAGTAGTTTATTTTTAAAAAATAAAATTTAAAATATTAGTGTATTTGATTTCCGAAAGTTAAAAAAGAAATAGTAAGAAATATATATTTCATTGAATGGATATATGAAACGTTTACTGGTGGAAGGCCGGCGGTTGTTTGCAAGACCGAGAAAAGGCTAGCAAGAATCGGGTCATTGTAGCGTATGCGCCTGTGAACATTCTCTTCAACAAGTTTGATTCCATTGCGGTGAAATGGTAAAAGTCAACCCCCTGCGATGTATATTTTCCTGTACAATCAATCAAAAAGCCAAATGATTTAGCATTATCTTTACATCTTGTTATTTTACAGATTTTATGTTTAGATCTTTTATGCTTGCTTTTCAAAAGGCCTGCAGGCAAGTGCACAAACAATGAATTCACCAGAACCACCAGAACCACCAGAACC 3’. The periCEN nucleotides are colored in grey. The CDEI nucleotides are colored in light orange. The CDEII nucleotides are colored in orange. The CDEIII nucleotides are dark orange. The linker DNA is colored in black.

For immunoblotting, silver stain, mass spectrometry and cryoEM sample preparation, the Dynabeads were washed with 10x bead volumes of Buffer L/0.175 (25 mM HEPES pH 7.6, 175 mM KGlutamate, 6 mM Mg(OAc)_2_, 0.1 mM EDTA pH 7.6, 0.5 mM EGTA-KOH, pH7.6, 0.1% NP-40, 15% glycerol) 5 times. The last 3 washes omitted DTT and phosphatase inhibitors. For immunoblots and silver stain, kinetochores were eluted with 0.5 mg/ml 3xFLAG peptide in Buffer H/0.15 lacking DTT and phosphatase inhibitors. For mass spectrometry, kinetochores were eluted from Dynabeads with 0.2% RapiGest (Waters Corporation, Milford MA) in 50 mM HEPES pH 8.0. For negative stain electron microscopy and cryoelectron microscopy, kinetochores were washed with 10x bead volumes of Buffer H/0.15 5 times (the last 3 washes omitting DTT and phosphatase inhibitors), followed by one wash in Buffer L/0.175-EM (25 mM HEPES pH 7.6, 175 mM KGlutamate, 6 mM Mg(OAc)_2_, 0.1 mM EDTA pH 7.6, 0.5 mM EGTA-KOH, pH7.6) and eluted in 20uL 0.5 mg/ml 3xFLAG (Genscript, Piscataway NJ) peptide at room temperature for 30 minutes.

### Negative stain EM

Eluates from Ame1-3XFLAG IP or assembly-IP were used for negative stain electron microscopy. For both samples, 4 uL of eluate was diluted with Buffer L and deposited on 400 mesh continuous carbon grid (Electron Microscopy Sciences) for 1 minute separately. The grids were washed twice with Milli-Q water and stained with 0.75% uranyl formate (Electron Microscopy Sciences) for 1 minute. The EM grids were air dried before loading into Talos L120C (ThermoFisher). The data was collected at 1.991Å/pixel and processed in cryoSPARC^44^.

### Analysis of correlation between CEN DNA and CCAN dimer

Whole cell extracts were prepared as described above. The extract was equally separated into three parts and 0 ng/mL, 607 ng/mL or 1200 ng/mL of 500 bp CEN3 DNA was added to each part and incubated at room temperature for 90 minutes. The assembled kinetochores were purified as described in “purification of kinetochore complexes”. The three eluates were used to prepare three batches of negative stain grids and processed individually. Particles from 2D classifications that represent both apo-CCAN and CCAN dimer were counted as total CCAN. The ratio between CCAN dimer/ total CCAN was calculated.

### CryoEM grid preparation and data collection

For Ame1-3XFLAG IP eluate grid preparation, 3uL undiluted eluate was applied on 1.2/1.3 gold grids (Au 300, Electron Microscopy Science) covered freshly with graphene oxide (REF) and incubated for 30 s at 4 °C with 100% relative humidity and vitrified using a Vitrobot (Mark IV, Thermo Fisher). The grids were imaged under a Glacios transmission electron microscope (Thermo Fisher Scientific) operated at 200kV. The microscope is equipped with a Gatan K3 summit direct detection camera (Gatan, Pleasanton). A total of 3,821 movies were collected at a pixel size of 1.384 Å. For each micrograph, a 50-frame movie stack was collected with total exposure at 50 e^−^/Å^2^.

For Ame1-3XFLAG assembly IP eluate grid preparation, 1.2/1.3 holey titanium grids (Single Particles) were plasma cleaned (plasma cleaner brand) for 30 s. 3 uL undiluted Ame1-FLAG assembly-IP eluate was applied on grids and incubated for 30 s at 4 °C with 100% relative humidity and vitrified using a Vitrobot (Mark IV, Thermo Fisher). The frozen grids were imaged under a Titan Krios G3 transmission electron microscope (Thermo Fisher Scientific) operated at 300 kV. The microscope is equipped with a Gatan K3 summit direct detection camera (Gatan, Pleasanton). A total of 12,069 movies were collected at a pixel size of 1.07 Å. For each micrograph, a 50-frame movie stack was collected with total exposure at 50 e^−^/Å^2^.

### CryoEM data processing

The two sets of collected movies were motion-corrected and dose-weighted in cryoSPARC seperately^44^. Contrast transfer function (CTF) corrected micrographs were used for blob picking and particles that belonged to well resolved 2D classes were used for topaz picking^59^ for second round particle picking. For Ame1-3XFLAG IP eluate samples, these particles only include apo-CCAN complexes and the particles were cleaned through multiple rounds of 2D classification and heterogenous refinement. A total of 88,088 particles was used for NU-refinement and a 3.9 Å density map was acquired. These particles include all four structures described in this paper. 744,531 particles were downsized and used for 2D classification. Classes that belong to apo-CCAN, Cbf1-CCAN-CEN, CCAN dimer, CBF3-CEN, and 40S, 60S and 80S ribosomes were used to run *ab initio* reconstructions, respectively. The remaining junk particles were also used to run ab initio reconstruction and separated into four classes. After obtaining the 10 initial models, we used them for the first round of heterogenous refinement. Particles that belong to the 6 good classes were used for a second round of heterogenous refinement using the 10 initial models. We ran heterogenous refinement for 6 rounds until the particles that belong to 6 good classes didn’t change dramatically between runs and then we re-extracted selected particles at pixel size 1.07 Å and box size 424×424. We ran NU-refinement on each initial model (Supplementary Fig. 2). To improve local resolution, we did local refinement on the apo-CCAN, CCAN dimer and CBF3-CEN complexes. The resolution for each density map was assessed using the gold-standard criterion of Fourier shell correlation (FSC), with a cutoff at 0.143^60,61^, between 2 half maps from 2 independent half-sets of data (Supplementary Fig. 2).

### Model building

#### a). CCAN monomer

The initial model of apo-CCAN that was previously solved (PDB: 6QLE) was roughly fit into a density map that was solved in this work. The Ame1-Okp1 arm fit well while there was a misfit on the HIK arm. The HIK arm structure was fit into a density map and the extra density present in our structure was modeled using coot^62^. The new model was refined with Phenix^63^ and the Ramachandran outliers were fixed in coot again. This procedure was repeated until there were no outliers present in the structure.

#### b). Cbf1-CCAN-CEN

Cbf1 and CEN structures were predicted with AlphaFold 3^38^ and used as an initial model. The HIK head and Cnn1-Wip1 were modeled using 8OVW. The CCAN model from a) was fit into a density map. All the models mentioned above were combined and refined using Phenix^63^ once. The CEN DNA didn’t fit into our density map, so we modeled the DNA in coot^62^. A similar approach was taken that we refined iteratively with coot and Phenix^64^ until the Ramachandran outliers were fixed.

#### c). CCAN dimer

Since the CCAN dimer density was relatively low resolution, we couldn’t decipher side chains in the model. Thus, we did a rigid body fit of two CCAN dimer models into the density. The HIK head model was also fit into the density map.

#### d). CBF3-CEN

The CBF3 and CEN were modeled using AlphaFold 3^38^. Two copies of CBF3 were docked into chimeric density, which combined both focused refined maps. A similar approach was taken as described in a) and b).

To predict the extra density sequence, we utilized a focused refined map and set the fulcrum at the center of the extra density to optimize resolution in this region. The new local map was used as input in Model-Angelo^45^ with the command: model_angelo build_no_seq -v map.mrc -o output. The output model sequence was used for an NCBI blast search for a similar protein peptide. The predicted sequence was aligned with Ndc10(579–639), and the new model was built by replacing the predicted residues into Ndc10 residues manually. The extra density model was then refined using the approach mentioned above.

### Immunoblot and silver stain analyses

For immunoblot analysis, cell lysates were prepared as described above. Protein samples were separated using pre-cast 4–12% Bis Tris Protein Gels (Thermo-Fisher Scientific, Waltham MA) for sodium dodecyl sulfate-polyacrylamide gel electrophoresis (SDS-PAGE) in MES buffer pH 7.0 (50 mM MES, 50 mM Tris, 0.1% SDS, 1 mM EDTA). For immunoblotting, a 0.45 μm nitrocellulose membrane (BioRad, Hercules CA) was used to transfer proteins from polyacrylamide gels. The antibodies used for immunoblotting were custom generated by Genscript (Piscataway, NJ) against recombinant proteins that were expressed and purified from *Escherichia coli* and then injected into rabbits^47,49^. Genscript affinity purified the antibodies using the recombinant proteins and the resulting antibodies were used at the following dilutions: α-Mif2 used at 1:3000; α-Ctf19 used at 1:5000; α-Okp1 used at 1:5000; α-Ame1 used at 1:5000; α-Chl4 used at 1:5000; α-Mtw1 used at 1:5000; α-Ndc80 used at 1:5000; α-Spc105 used at 1:5000. Genscript services were also used to generate a FLAG antibody and V5 antibody which were used at 1:10,000^49^. The antibodies against Pgk1 and H2A were purchased from Invitrogen (4592560 and 39235) and used at 1:10,000 and 1:2000. α-Ndc10^37^ and α-Cse4^64^ were used at 1:5000 and 1:500. The secondary antibodies used were a sheep α-mouse antibody conjugated to horseradish peroxidase (HRP) (GE Life sciences, Marlborough MA) at a 1:10,000 dilution or a donkey α-rabbit antibody conjugated to HRP (GE Life sciences, Marlborough MA) at a 1:10,000 dilution. Antibodies were detected using the Super Signal West Dura Chemiluminescent Substrate (Thermo-Fisher Scientific, Waltham MA). For analysis by silver stain, the gels were stained with Silver Quest Staining Kit according to manufacturer’s instructions (Invitrogen, Waltham MA).

### Serial dilution assay

The designated *S. cerevisiae* strains were grown in YPD overnight. 1mL culture was spun down for each of the strains and the pellets were resuspended in 1mL YEP, respectively. Then the cell concentration was measured on a spectrophotometer (Bio-Rad), and cells were diluted to OD600 = 1.0. Next, serial dilutions (1:5) were made in water in a 96-well plate and the wells were then spotted onto YPD or YEP plates supplemented with 2% galactose and 2% raffinose. Plates were grown at the indicated temperatures for 1–3 days prior to imaging.

### Fiber-seq

In-house Hia5 preparation and yeast Fiber-seq were performed as described^48,49^. In brief, 10 ml cultures of wild-type (SBY3) and *ndc10*^*6A*^ (SBY24368) yeast cells were grown in YPD medium to mid-log phase and collected by centrifugation. Cells were washed once with cold dH_2_O and resuspended in cold KPO4/Sorbitol buffer (1 M sorbitol, 50 mM potassium phosphate pH 7.5, 5 mM EDTA pH 8.0) supplemented with 0.167% β-mercaptoethanol. Spheroplasts were generated by adding Zymolyase T100 (0.15 μg/mL final concentration; Amsbio) and incubating at 23 °C for ~15 min on a roller drum. Spheroplasts were pelleted at 160 g for 8 min at 4 °C, washed twice with cold 1 M Sorbitol, and resuspended in 58 uL of Buffer A (1 M Sorbitol, 15 mM Tris-HCl pH 8.0, 15 mM NaCl, 60 mM KCl, 1 mM EDTA pH 8.0, 0.5 mM EGTA pH 8.0, 0.5 mM Spermidine, 0.075% IGEPAL CA-630). Spheroplasts were treated with 1 μL of Hia5 MTase (200U) and 1.5 μL of 32 mM S-adenosylmethionine (NEB) for 10 min at 25 °C. The reaction was stopped by addition of 3 μL of 20% SDS (1% final concentration) and high molecular weight DNA was purified using the Promega Wizard^®^ HMW DNA extraction kit (A2920). Circular consensus sequence reads were generated from raw PacBio subread files and processed as previously described^65^. Reads were aligned to the April 2011 *sacCer3* yeast reference genome, and nucleosomes were identified using default fibertools parameters^66^. To control for minor technical differences in the overall m6A methylation rate between each sample, reads were subsetted to normalize the per-read methylation distribution across samples (https://github.com/StergachisLab/match-distribution). Fibers overlapping with the center of each sixteen centromeres were extracted. The nucleosome density was calculated by counting the number of nucleosomes that overlap with each base pair of the region of interest divided by the number of fibers overlapping with that position. The averaged nucleosome density profiles across all sixteen centromeres are shown in Fig. 4d, and an individual profile for CEN3 is shown in Fig. 4e and 4g.

### Reconstituted Ndc10^D3-D5^ purification

Plasmids derived from pET28b were used for expressing WT or mutant Ndc10^D3-D5^ with an N-terminal 6xHIS tag. The plasmids were transformed into Rosetta2 competent cells and single colonies were inoculated in terrific broth with 25 ug/mL ampicillin to OD_600_=0.5. The cultures were then induced with 0.1 mM IPTG at 18 °C overnight before being spun down at 5000g for 10 mins. The cell pellet was resuspended in Buffer H/1.0 (25 mM HEPES pH 8.0, 1M KCl, 2 mM MgCl_2_, 0.1 mM EDTA pH 8.0, 0.1% NP-40, 15% glycerol) supplemented with 2 mM PMSF, EDTA-free protease cocktail and 10 mM imidazole. The cells were sonicated at 50% amplitude for 4 minutes with 1 second on and 1 second off. The supernatant was clarified by centrifugation at 13,000 g for 30 mins before applying to a 2 mL Talon cobalt resin and mixing for 1 hr in the cold room. The resin was then washed with 40 mL Buffer H/0.5 (25 mM HEPES pH 8.0, 500 mM KCl, 2 mM MgCl_2_, 0.1 mM EDTA pH 8.0, 0.1% NP-40, 15% glycerol, 20 mM imidazole) and eluted with 150 mM imidazole in Buffer H/0.15 (25 mM HEPES pH 8.0, 150 mM KCl, 2 mM MgCl_2_, 0.1 mM EDTA pH 8.0, 15% glycerol, 150 mM imidazole). The purified eluates were verified using SDS-PAGE (Supplementary Fig. 7a).

### Mass photometry

Mass photometry experiments were performed using the TwoMP system by Refey^67^. Movies were collected with Acquire2024 R1.1 and data was analyzed using Discover 2024 R1.0. The autofocus function was used to find the focus plane using 19 μl of cold modified buffer H 0.15 (25 mM HEPES pH 8.0, 150 mM KCl, 2 mM MgCl_2_, 0.1 mM EDTA pH 8.0, 15% glycerol) on uncoated glass slides (Refeyn). Thyroglobulin monomer and dimer peaks, conalbumin and aldolase were used as standards. Purified WT or mutant Ndc10^D3-D5^ fragments were diluted to 20 nM in cold modified Buffer H/0.15 and applied as droplet for measurement. Representative histograms of WT and mutants are shown in Supplementary Fig. 7b.

### TIRFM imaging and analysis

TIRFM assay to detect *de novo* kinetochore assembly at the single molecule level was performed as previously described^46,47^. Briefly, all images were captured using a Nikon TE-2000 inverted RING-TIRF microscope. Images were acquired at a resolution of 512 × 512 pixels with a pixel size of 0.11 μm/pixel at a readout speed of 10 MHz. Atto-647-labeled CEN DNAs were excited at 640 nm for 300 ms, GFP-tagged proteins at 488 nm for 200 ms. Images were analyzed with the CellProfiler (4.2.6) to assess colocalization and quantify signals between the DNA channel (647 nm) and GFP channel (488nm). Results were processed and visualized using FIJI (https://imagej.net/software/fij). For photobleaching assays, images in the 488 nm channel were acquired every 100 ms. The DNA-channel (647 nm) was only imaged for the initial frame throughout the movie. Photobleaching steps were analyzed using MATLAB to extract intensity traces of the 488 nm channel, and step count was performed using the same method as described^49,68^.

### Fluorescent microscopy of fixed yeast cells

WT or mutant *ndc10* strains with Mtw1-mYPet^51^ and Spc110-mTurquoise2^52^ were inoculated starting from OD_600_=0.4. To synchronize cells in G1 phase, 1ug/mL α factor was added to the culture and inoculated for 3 hours. 500 uM auxin was added 20 minutes before releasing cells to degrade Ndc10-AID. The cells were then washed two times with YEP containing 500 uM auxin and released for 105 mins before fixation. The cells were fixed with 3.7% formaldehyde at room temperature for 10 mins. To visualize the DNA, fixed cells were incubated with 1 ug/mL DAPI in 1.2M Sorbitol, 1% Triton, 0.1M potassium phosphate for 10 mins at room temperature. The stained cells were then pelleted and resuspended in 1.2M Sorbitol, 1% Triton, 0.1M potassium phosphate before imaging.

Fixed cell images were acquired on a Deltavision Ultra deconvolution high-resolution microscope equipped with a 60x/1.42 PlanApo N oil-immersion objective (Olympus). equipped with a 16-bit sCMOS detector. On both microscopes, cells were imaged in Z-stacks through the entire cell using 0.5 μm steps. All images were deconvolved using standard settings. All the images were projected for Z stack max intensity and normalized using FIJI. Cell stage was identified using Spc110-mTurquoise2 signals marking the two spindle pole bodies.

For Mtw1-YPet signal counting, only cells with visible spindle pole body signals were counted. The cells with an Mtw1 signal as strong as the WT strains were assigned as a strong signal. The cells with much weaker Mtw1 signal were assigned as a weak signal. The cells with only a diffuse Mtw1 signal were assigned as no signal. For all measured strains, 200 cells were counted and the experiment was repeated 3 times for a two-tailed t test to calculate the significance difference.

To quantify the DNA distribution in daughter cells, only anaphase cells with fully separated spindle pole bodies were counted. Cells with DAPI signals that were evenly distributed into daughter cells were counted as normal segregation while DAPI signals that were not evenly distributed were counted as chromosome missegregation. The experiments were repeated three times on three batches of cells.

## Data Availability

Mass spectrometry data generated in this study is available through Mass Spectrometry Interactive Virtual Environment (MassIVE, University of California San Diego) with the link https://massive.ucsd.edu/ProteoSAFe/dataset.jsp?task=a50a708407634efc951e1828867bd934. The density map and model of apo-CCAN are accessible with code EMD-75131 from EM Databank and 10FI from Protein Data Bank. The density map and model of CCAN dimer are accessible with code EMD-75213 from EM Databank and 10JC from Protein Data Bank. The density map and model of Cbf1-CCAN-CEN are accessible with code EMD-75095 from EM Databank and 10DQ from Protein Data Bank. The density map and model of CBF3-CEN are accessible with code EMD-75107 from EM Databank and 10EH from Protein Data Bank. The plasmids used in this study can be provided when requested.

## References

[1] Cheeseman I. M. The kinetochore. Cold Spring Harbor Perspectives in Biology 6, a015826(2014). 24984773 10.1101/cshperspect.a015826 PMC4067989

[2] Biggins S. The composition, functions, and regulation of the budding yeast kinetochore. Genetics 194, 817–846 (2013). 23908374 10.1534/genetics.112.145276 PMC3730914

[3] Cleveland D. W., Mao Y. & Sullivan K. F. Centromeres and kinetochores: from epigenetics to mitotic checkpoint signaling. Cell 112, 407–421 (2003). 12600307 10.1016/s0092-8674(03)00115-6

[4] Torras-Llort M., Moreno-Moreno O. & Azorín F. Focus on the centre: the role of chromatin on the regulation of centromere identity and function. EMBO J 28, 2337–2348 (2009). 19629040 10.1038/emboj.2009.174 PMC2722248

[5] Allshire R. C. & Karpen G. H. Epigenetic regulation of centromeric chromatin: old dogs, new tricks? Nat Rev Genet 9, 923–937 (2008). 19002142 10.1038/nrg2466 PMC2586333

[6] Musacchio A. & Desai A. A molecular view of kinetochore assembly and function. Biology 6, 5 (2017). 28125021 10.3390/biology6010005 PMC5371998

[7] Westhorpe F. G. & Straight A. F. Functions of the centromere and kinetochore in chromosome segregation. Current Opinion in Cell Biology 25, 334–340 (2013). 23490282 10.1016/j.ceb.2013.02.001 PMC3687001

[8] Nagpal H. & Fukagawa T. Kinetochore assembly and function through the cell cycle. Chromosoma 125, 645–659 (2016). 27376724 10.1007/s00412-016-0608-3

[9] Yamagishi Y., Sakuno T., Goto Y. & Watanabe Y. Kinetochore composition and its function: lessons from yeasts. FEMS Microbiol Rev 38, 185–200 (2014). 24666101 10.1111/1574-6976.12049

[10] Pesenti M. E., Weir J. R. & Musacchio A. Progress in the structural and functional characterization of kinetochores. Current Opinion in Structural Biology 37, 152–163 (2016). 27039078 10.1016/j.sbi.2016.03.003

[11] Furuyama S. & Biggins S. Centromere identity is specified by a single centromeric nucleosome in budding yeast. Proc. Natl. Acad. Sci. U.S.A. 104, 14706–14711 (2007). 17804787 10.1073/pnas.0706985104 PMC1976213

[12] Clarke L. & Carbon J. Isolation of a yeast centromere and construction of functional small circular chromosomes. Nature 287, 504–509 (1980). 6999364 10.1038/287504a0

[13] Clarke L. Centromeres: proteins, protein complexes, and repeated domains at centromeres of simple eukaryotes. Current Opinion in Genetics & Development 8, 212–218 (1998). 9610412 10.1016/s0959-437x(98)80143-3

[14] Díaz-Ingelmo O., Martínez-García B., Segura J., Valdés A. & Roca J. DNA topology and global architecture of point centromeres. Cell Reports 13, 667–677 (2015). 26489472 10.1016/j.celrep.2015.09.039

[15] Wilmen A., Pick H., Niedenthal R. K., Sen-Gupta M. & Hegemann J. H. The yeast centromere CDEI/Cpf1 complex: differences between *in vitro* binding and *in vivo* function. Nucl Acids Res 22, 2791–2800 (1994). 8052535 10.1093/nar/22.14.2791 PMC308249

[16] Niedenthal R., Stoll R. & Hegemann J. H. *In vivo* characterization of the *Saccharomyces cerevisiae* centromere DNA element I, a binding site for the helix-loop-helix protein CPF1. Molecular and Cellular Biology 11, 3545–3553 (1991). 2046668 10.1128/mcb.11.7.3545-3553.1991 PMC361097

[17] Lechner J. & Carbon J. A 240 kd multisubunit protein complex, CBF3, is a major component of the budding yeast centromere. Cell 64, 717–725 (1991). 1997204 10.1016/0092-8674(91)90501-o

[18] Jehn B., Niedenthal R. & Hegemann J. H. *In vivo* analysis of the *Saccharomyces cerevisiae* centromere CDEIII sequence: requirements for mitotic chromosome segregation. Mol. Cell. Biol. 11, 5212–5221 (1991). 1922041 10.1128/mcb.11.10.5212-5221.1991 PMC361563

[19] Meluh P. B. & Koshland D. Budding yeast centromere composition and assembly as revealed by *in vivo* cross-linking. Genes Dev. 11, 3401–3412 (1997). 9407032 10.1101/gad.11.24.3401 PMC524546

[20] Baker R. E. & Rogers K. Genetic and genomic analysis of the AT-Rich centromere DNA element II of *Saccharomyces cerevisiae*. Genetics 171, 1463–1475 (2005). 16079225 10.1534/genetics.105.046458 PMC1350974

[21] Dendooven T. Cryo-EM structure of the complete inner kinetochore of the budding yeast point centromere. Sci. Adv. 9, eadg7480 (2023). 10.1126/sciadv.adg7480 PMC10381965 37506202

[22] Zhou B.-R. Atomic resolution cryo-EM structure of a native-like CENP-A nucleosome aided by an antibody fragment. Nat Commun 10, 2301 (2019). 31127102 10.1038/s41467-019-10247-4 PMC6534667

[23] Yan K. Structure of the inner kinetochore CCAN complex assembled onto a centromeric nucleosome. Nature 574, 278–282 (2019). 31578520 10.1038/s41586-019-1609-1 PMC6859074

[24] Doğan D. CENP-A nucleosome is a sensitive allosteric scaffold for DNA and chromatin factors. Journal of Molecular Biology 433, 166789 (2021). 33387534 10.1016/j.jmb.2020.166789

[25] Leber V., Nans A. & Singleton M. R. Structural basis for assembly of the CBF3 kinetochore complex. The EMBO Journal 37, 269–281 (2018). 29212814 10.15252/embj.201798134 PMC5771398

[26] Lechner J. A zinc finger protein, essential for chromosome segregation, constitutes a putative DNA binding subunit of the *Saccharomyces cerevisiae* kinetochore complex, Cbf3. The EMBO Journal 13, 5203–5211 (1994). 7957085 10.1002/j.1460-2075.1994.tb06851.x PMC395469

[27] Strunnikov A. V., Kingsbury J. & Koshland D. Cep3 encodes a centromere protein of *Saccharomyces cerevisiae*. J Cell Biol 128, 749–760 (1995). 7876302 10.1083/jcb.128.5.749 PMC2120391

[28] Purvis A. & Singleton M. R. Insights into kinetochore–DNA interactions from the structure of Cep3Δ. EMBO Reports 9, 56–62 (2008). 18064045 10.1038/sj.embor.7401139 PMC2246632

[29] Espelin C. W., Kaplan K. B. & Sorger P. K. Probing the architecture of a simple kinetochore using DNA–protein crosslinking. The Journal of Cell Biology 139, 1383–1396 (1997). 9396745 10.1083/jcb.139.6.1383 PMC2132615

[30] Bouck D. C. & Bloom K. S. The kinetochore protein Ndc10p is required for spindle stability and cytokinesis in yeast. Proc. Natl. Acad. Sci. U.S.A. 102, 5408–5413 (2005). 15809434 10.1073/pnas.0405925102 PMC556225

[31] Guan R. Structural and dynamic mechanisms of CBF3-guided centromeric nucleosome formation. Nat Commun 12, 1763 (2021). 33741944 10.1038/s41467-021-21985-9 PMC7979930

[32] Yan K., Zhang Z., Yang J., McLaughlin S. H. & Barford D. Architecture of the CBF3–centromere complex of the budding yeast kinetochore. Nat Struct Mol Biol 25, 1103–1110 (2018). 30478265 10.1038/s41594-018-0154-1 PMC6292502

[33] Hinshaw S. M. & Harrison S. C. The structure of the Ctf19c/CCAN from budding yeast. eLife 8, e44239 (2019). 30762520 10.7554/eLife.44239 PMC6407923

[34] Akiyoshi B. Tension directly stabilizes reconstituted kinetochore-microtubule attachments. Nature 468, 576–579 (2010). 21107429 10.1038/nature09594 PMC3108429

[35] Akiyoshi B. The Mub1/Ubr2 ubiquitin ligase complex regulates the conserved Dsn1 kinetochore protein. PLoS Genet 9, e1003216 (2013). 23408894 10.1371/journal.pgen.1003216 PMC3567142

[36] Gupta A., Evans R. K., Koch L. B., Littleton A. J. & Biggins S. Purification of kinetochores from the budding yeast *Saccharomyces cerevisiae*. in Methods in Cell Biology vol. 144 349–370 (Elsevier, 2018). 29804677 10.1016/bs.mcb.2018.03.023 PMC6205221

[37] Lang J., Barber A. & Biggins S. An assay for *de novo* kinetochore assembly reveals a key role for the CENP-T pathway in budding yeast. eLife 7, e37819 (2018). 30117803 10.7554/eLife.37819 PMC6097842

[38] Abramson J. Accurate structure prediction of biomolecular interactions with AlphaFold 3. Nature 630, 493–500 (2024). 38718835 10.1038/s41586-024-07487-w PMC11168924

[39] Donovan B. T. Basic helix-loop-helix pioneer factors interact with the histone octamer to invade nucleosomes and generate nucleosome-depleted regions. Molecular Cell 83, 1251–1263.e6 (2023). 36996811 10.1016/j.molcel.2023.03.006 PMC10182836

[40] Bechert T. All 16 centromere DNAs from *Saccharomyces cerevisiae* show DNA curvature. Nucleic Acids Research 27, 1444–1449 (1999). 10037804 10.1093/nar/27.6.1444 PMC148336

[41] Hinshaw S. M. & Harrison S. C. An Iml3-Chl4 heterodimer links the core centromere to factors required for accurate chromosome segregation. Cell Reports 5, 29–36 (2013). 24075991 10.1016/j.celrep.2013.08.036 PMC3888643

[42] Pietrasanta L. I. Probing the *Saccharomyces cerevisiae* centromeric DNA (CEN DNA)–binding factor 3 (CBF3) kinetochore complex by using atomic force microscopy. Proc. Natl. Acad. Sci. U.S.A. 96, 3757–3762 (1999). 10097110 10.1073/pnas.96.7.3757 PMC22367

[43] Cho U.-S. & Harrison S. C. Ndc10 is a platform for inner kinetochore assembly in budding yeast. Nat Struct Mol Biol 19, 48–55 (2012). 10.1038/nsmb.2178 PMC3252399 22139014

[44] Punjani A., Rubinstein J. L., Fleet D. J. & Brubaker M. A. cryoSPARC: algorithms for rapid unsupervised cryo-EM structure determination. Nat Methods 14, 290–296 (2017). 28165473 10.1038/nmeth.4169

[45] Jamali K. Automated model building and protein identification in cryo-EM maps. Nature 628, 450–457 (2024). 38408488 10.1038/s41586-024-07215-4 PMC11006616

[46] Hu C., Popchock A. R., Latino A. A., Asbury C. L. & Biggins S. Direct observation of interdependent and hierarchical kinetochore assembly on individual centromeres. Nucleic Acids Research 53, gkaf1038 (2025). 10.1093/nar/gkaf1038 PMC12526914 41099706

[47] Popchock A. R., Larson J. D., Dubrulle J., Asbury C. L. & Biggins S. Direct observation of coordinated assembly of individual native centromeric nucleosomes. The EMBO Journal 42, e114534 (2023). 37469281 10.15252/embj.2023114534 PMC10476280

[48] Stergachis A. B., Debo B. M., Haugen E., Churchman L. S. & Stamatoyannopoulos J. A. Single-molecule regulatory architectures captured by chromatin fiber sequencing. Science 368, 1449–1454 (2020). 32587015 10.1126/science.aaz1646

[49] Popchock A. R. Stable centromere association of the yeast histone variant Cse4 requires its essential N-terminal domain. EMBO J 44, 1488–1511 (2025). 39809842 10.1038/s44318-024-00345-5 PMC11876619

[50] Yesbolatova A. The auxin-inducible degron 2 technology provides sharp degradation control in yeast, mammalian cells, and mice. Nat Commun 11, 5701 (2020). 33177522 10.1038/s41467-020-19532-z PMC7659001

[51] Nguyen A. W. & Daugherty P. S. Evolutionary optimization of fluorescent proteins for intracellular FRET. Nat Biotechnol 23, 355–360 (2005). 15696158 10.1038/nbt1066

[52] Goedhart J. Structure-guided evolution of cyan fluorescent proteins towards a quantum yield of 93%. Nat Commun 3, 751 (2012). 22434194 10.1038/ncomms1738 PMC3316892

[53] Xiao H. Nonhistone Scm3 binds to AT-Rich DNA to organize atypical centromeric nucleosome of budding yeast. Molecular Cell 43, 369–380 (2011). 21816344 10.1016/j.molcel.2011.07.009 PMC6993184

[54] Xiao H. Molecular basis of CENP-C association with the CENP-A nucleosome at yeast centromeres. Genes Dev. 31, 1958–1972 (2017). 29074736 10.1101/gad.304782.117 PMC5710141

[55] Yu G., Li K. & Jiang W. Antibody-based affinity cryo-EM grid. Methods 100, 16–24 (2016). 26804563 10.1016/j.ymeth.2016.01.010 PMC4848123

[56] Chio U. S. Functionalized graphene-oxide grids enable high-resolution cryo-EM structures of the SNF2h-nucleosome complex without crosslinking. Nat Commun 15, 2225 (2024). 38472177 10.1038/s41467-024-46178-y PMC10933330

[57] Joglekar A. P., Salmon E. D. & Bloom K. S. Counting kinetochore protein numbers in budding yeast using genetically encoded fluorescent proteins. in Methods in Cell Biology vol. 85 127–151 (Elsevier, 2008). 18155462 10.1016/S0091-679X(08)85007-8 PMC2892121

[58] Rose M. David., Winston F. Marshall. & Hieter Philip. Methods in Yeast Genetics : A Laboratory Course Manual. (Cold Spring Harbor Laboratory Press, Cold Spring Harbor, N.Y, 1990).

[59] Bepler T. Positive-unlabeled convolutional neural networks for particle picking in cryo-electron micrographs. Nat Methods 16, 1153–1160 (2019). 31591578 10.1038/s41592-019-0575-8 PMC6858545

[60] Scheres S. H. W. & Chen S. Prevention of overfitting in cryo-EM structure determination. Nat Methods 9, 853–854 (2012). 22842542 10.1038/nmeth.2115 PMC4912033

[61] Henderson R. Outcome of the first electron microscopy validation task force meeting. Structure 20, 205–214 (2012). 22325770 10.1016/j.str.2011.12.014 PMC3328769

[62] Emsley P., Lohkamp B., Scott W. G. & Cowtan K. Features and development of Coot. Acta Crystallogr D Biol Crystallogr 66, 486–501 (2010). 20383002 10.1107/S0907444910007493 PMC2852313

[63] Afonine P. V. Real-space refinement in PHENIX for cryo-EM and crystallography. Acta Crystallogr D Struct Biol 74, 531–544 (2018). 29872004 10.1107/S2059798318006551 PMC6096492

[64] Pinsky B. A., Tatsutani S. Y., Collins K. A. & Biggins S. An Mtw1 complex promotes kinetochore biorientation that is monitored by the Ipl1/Aurora protein kinase. Developmental Cell 5, 735–745 (2003). 14602074 10.1016/s1534-5807(03)00322-8

[65] Vollger M. R. Synchronized long-read genome, methylome, epigenome and transcriptome profiling resolve a mendelian condition. Nat Genet 57, 469–479 (2025). 39880924 10.1038/s41588-024-02067-0 PMC12077378

[66] Jha A. DNA-m6A calling and integrated long-read epigenetic and genetic analysis with fibertools. Genome Res. 34, 1976–1986 (2024). 38849157 10.1101/gr.279095.124 PMC11610455

[67] Young G. Quantitative mass imaging of single biological macromolecules. Science 360, 423–427 (2018). 29700264 10.1126/science.aar5839 PMC6103225

[68] Chen Y., Deffenbaugh N. C., Anderson C. T. & Hancock W. O. Molecular counting by photobleaching in protein complexes with many subunits: best practices and application to the cellulose synthesis complex. MBoC 25, 3630–3642 (2014). 25232006 10.1091/mbc.E14-06-1146 PMC4230622

